# Medial Femoral Condyle Cyst in Severe Varus Knee Osteoarthritis: A Finding Prior to Primary Total Knee Arthroplasty

**DOI:** 10.7759/cureus.45372

**Published:** 2023-09-16

**Authors:** Muath Alqahtani, Ahmed Jalal, Mamdouh Masri

**Affiliations:** 1 Orthopedic Surgery, King Fahad General Hospital, Jeddah, SAU; 2 Orthopedic Surgery, King Abdulaziz Hospital, Makkah, SAU; 3 Orthopedic Surgery, Dr. Soliman Fakeeh Hospital, Jeddah, SAU

**Keywords:** osteoarthritis, arthroplasty, cyst, knee, arthritis

## Abstract

Knee osteoarthritis (OA) often results in subchondral bone cysts (SBCs), which were initially identified as a concentric arrangement of trabeculae surrounding an enlarged marrow space on plain radiographs. Although the Anderson Orthopedic Research Institute (AORI) classification is commonly used, it lacks quantitative measures and is based on radiographs, which can underestimate the actual bone defect. There is a need for a more comprehensive classification system to achieve accurate preoperative planning for bone defect management. A 74-year-old male presented, complaining of bilateral knee pain that began seven years ago. Non-operative management failed to relieve his symptoms despite his ideal BMI of 23.6. Initial radiographs revealed severe bilateral OA (Kellgren and Lawrence grade 4) with a suspected cyst occupying the medial femoral condyle. A CT scan of the right knee confirmed the presence of a cyst in the medial femoral condyle. The authors believe that patients with severe knee OA (Kellgren and Lawrence grade 3 or 4) should not simply be treated as having a sequel of knee arthritis. Instead, a CT scan should be conducted to confirm the size and extent of any cyst.

## Introduction

The incidence of knee osteoarthritis (OA) in the United States is around 240 persons per 100,000 per year. The worldwide prevalence of radiographically confirmed knee OA is approximately 3.8% overall, increasing with age to more than 10% in the population older than 60 years [1]. Subchondral bone cysts (SBCs) were initially understood as concentric arrangements of trabeculae around an enlarged marrow space on plain radiographs. On MRI, SBCs display well-defined rounded areas with fluid-like signal intensity. MRI is more sensitive than radiography for detecting small SBCs, identifying them in up to 57% of OA patients [2]. Different classification systems for bone defects in primary and revision total knee arthroplasty (TKA) have been established. The Dorr classification is designed to classify tibial defects in primary and revision cases in terms of peripheral or central defects without defining the defect size [3]. On the other hand, the Rand classification offers four types of femoral defects (minimal, moderate, extensive, and massive) based on depth and the area of condylar involvement as a numeric percentage but provides no guided treatment [4]. Although the Anderson Orthopedic Research Institute (AORI) classification is commonly used and provides guidance for surgical options, it lacks a quantitative measure and is based on radiographs, which can underestimate the actual bone defect [5]. Qiu YY et al. stated in their review article about revision TKA bone defects that "No single system can perfectly predict intra-operative bone defects from preoperative radiographs." An ideal classification system should aid in accurate preoperative planning for bone defect management. The intra- and inter-observer errors should be assessed to determine the accuracy and reproducibility of the classification systems [6]. Several solutions have emerged to address bone loss in arthritic knees based on the degree of metaphyseal involvement and containment. Proper and adequate planning is essential, along with an intraoperative assessment of the extent of bone loss on both the tibial and femoral sides. Mancuso F et al. found in their review that the Anderson Orthopedic Research Institute (AORI) classification is commonly used, and the choice to address bone loss depends on the size, location, and host bone quality. "Fill and Fix" is the cornerstone for addressing bone loss during TKA, where filling the bone defect with impacted bone graft or cement is followed by addressing the containment of the defect with either metaphyseal or diaphyseal fixation implants [7].

In this report, we present a case of severe bilateral knee OA with bony loss in the right knee's tibial plateau and a suspected subchondral cyst in the medial femoral condyle.

## Case presentation

**Figure 1:**
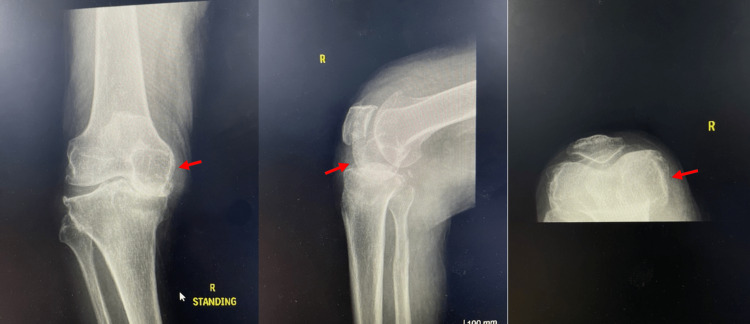
Anterior to posterior, lateral and skyline merchant view of the right knee showing a double shadowing (red arrow) on the right femoral condyle. The decreased bone density compared to the lateral condyle raises suspicion of an underlying bony defect.

A 74-year-old male with no known comorbidities presented to the clinic with a long-standing complaint of bilateral knee pain, more pronounced in the left knee. The pain began seven years ago and was manageable with analgesia and non-operative treatments until it became severe in the past year. Despite his ideal BMI of 23.6, his daily activities have been significantly impacted. His walking distance has reduced such that pain occurs after just two minutes of pain-free walking, and he experiences bedtime pain at the end of the day. The patient has tried multiple non-operative management modalities for knee OA, including physical therapy and bilateral knee intra-articular injections (hyaluronic acid), but they have not alleviated his symptoms. At the time of his presentation, he relied on a wheelchair for community ambulation. Upon physical examination, he exhibited moderate, semi-fixed bilateral genu varum, more pronounced in the right knee joint, accompanied by swelling and atrophy of the quadriceps and hamstrings. This atrophy underscored the severe disuse and disability sequelae of long-standing knee OA. During active and passive range of motion (ROM) testing, he lacked 5 degrees of terminal knee extension and exhibited crepitus throughout the knee ROM, with a maximum flexion of 130 degrees. The varus-valgus stress test was positive for the varus stress test, indicative of prolonged genu varus knee deformity and laxity of the lateral knee ligamentous-capsular complex. The Knee Society Score (KSS) calculation revealed a score of 53, classified as 'Poor'. Initial radiographs revealed severe bilateral OA (Kellgren and Lawrence grade 4). This report highlights the findings in the right knee (Figure 1), which show severe right knee OA characterized by obliteration of the medial joint space, subchondral sclerosis, osteophytes, and a suspicion of a medial femoral condyle occupying cyst, indicated by sclerotic demarcation of increased radiolucency in the medial femoral condyle. Additionally, there is evidence of tibial-sided medial bone loss and plateau depression.

**Figure 2:**
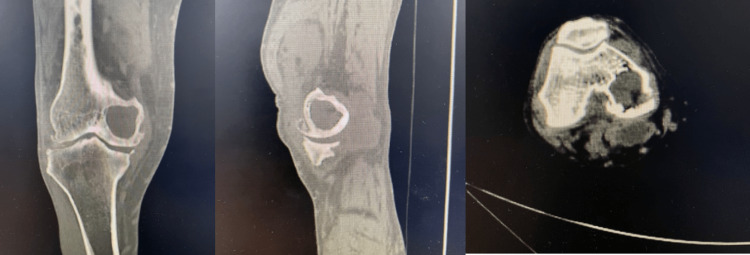
CT scan of the right knee showing bony cyst occupying medial femoral condyle.

A CT scan was chosen as the next step to fully understand the presence and size of the femoral condyle bone cyst, as well as to assess the degree of bone loss on both the femoral and tibial sides in planning for total knee arthroplasty. The right knee CT scan (Figure 2) revealed a medial condyle-occupying cyst with a posteromedial cortical perforation of the cyst, in addition to severe tricompartmental OA, medial tibial plateau bone loss, and depression. Although the patient ultimately chose not to undergo surgery, the initial plan was to proceed with total knee replacement (TKR), utilizing diaphyseal fixation of the construct with stemmed implants. The decision to use bone grafting or cement versus porous-coated metaphyseal augmentation would be based on the intra-operative assessment of the extent of bony loss on the tibial and femoral sides (whether the defect is contained or uncontained).

## Discussion

**Figure 3:**
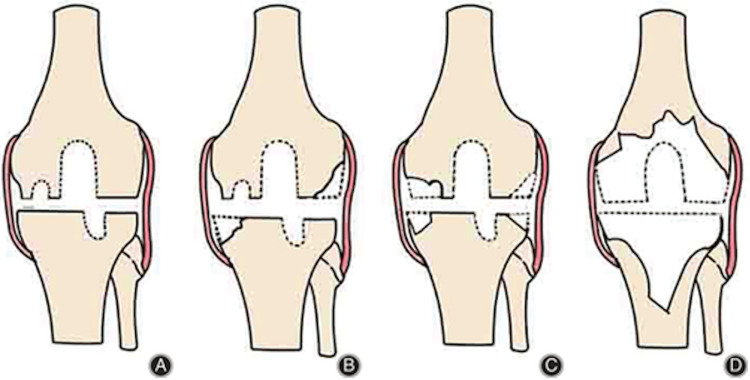
AORI classification: (A) Type I (intact metaphysis with minor defects), (B) Type IIA (defects in the metaphysis and one femoral condyle or tibial plateau), (C) Type IIB (defects in more than one metaphyseal region), and (D) Type III (bone loss compromising a major portion of the condyle or plateau).

Bony defects in primary TKA may catch surgeons off-guard. Although CT scans have no clear indications, some surgeons may require a CT in cases of difficult arthroplasty. While the AORI classification (Figure 3) is the most commonly used and provides a guided plan, it lacks a quantitative measure and is radiograph-based, leading to an underestimation of the actual bone defect [5].

A CT scan is a valuable investigative modality for suspected SBCs resulting from physiological remodeling due to long-standing OA of the knee. Bousson V et al. developed an image analysis software called the Medical Image Analysis Framework (MIAF)-Knee. It presents CT imaging of the subchondral bone in knee osteoarthritis and provides a brief introduction to the basic technical aspects of MIAF-Knee, as well as preliminary results obtained in patients with knee osteoarthritis compared to control subjects. This suggests that in mineralized tissues such as subchondral bone in cases of knee OA, the CT technique offers a valuable contribution [8].

Preoperative preparation and planning for a patient suffering from severe knee OA requires addressing the degree of bone loss, as well as the location and size of the SBC if present. This allows the surgeon to fully comprehend the patient's requirements for instrumentation, including augmentation based on the size and containment of the bony defect. In their review, odríguez-Merchán EC et al. mentioned various techniques to address bone defects. Small to moderate defects can be managed by cement and screws, impacted bone grafting, metal augment, and stemmed implants. However, severe defects are problematic and may require highly porous metal augments such as cones and sleeves [9]. Roach RP et al. found in their review a comparable similarity between sleeves and cones in terms of aseptic loosening. In contrast, a nearly doubled reoperation rate was found with Tantalum cones [10].

## Conclusions

The aim of TKA is to restore the patient's pain-free lifestyle along with functional level restoration. Addressing the element of bone loss prior to knee arthroplasty is an essential part of planning for complex primary TKR to avoid intraoperative complications due to a lack of instrumentation, special implants, or bone grafts. This can be achieved by complete preoperative imaging and planning, with a low threshold for CT if significant bone loss is anticipated, as well as having revision-type implants available to achieve the best postoperative outcomes. While bone loss may seem like a usual sequel to knee OA, in reality, the severity of bone loss varies among patients. Significant bone loss is unusual in knee OA unless very advanced OA exists. Nevertheless, authors believe that bone loss (including bone cysts) in patients with severe knee OA (Kellgren and Lawrence grade 3 or 4) should not be viewed as a sequel to knee OA without comprehensive preoperative assessment. It should be addressed with a CT scan to confirm the size and extent of the cyst and to assess the TKR design that fits appropriately with the patient's condition, considering cost-efficiency. It is not prudent to proceed with a revision design for a simple bone cyst or vice versa. A higher level of evidence is needed to consider (1) evaluating the current preoperative knee bone loss classification and its application and (2) establishing clinical criteria/algorithms for patients with advanced OA to determine whether a preoperative CT scan is indicated to address bone loss prior to TKR.
